# Effects on Bone and Muscle upon Treadmill Interval Training in Hypogonadal Male Rats

**DOI:** 10.3390/biomedicines11051370

**Published:** 2023-05-05

**Authors:** Ioannis Stratos, Ingmar Rinas, Konrad Schröpfer, Katharina Hink, Philipp Herlyn, Mario Bäumler, Tina Histing, Sven Bruhn, Brigitte Müller-Hilke, Michael D. Menger, Brigitte Vollmar, Thomas Mittlmeier

**Affiliations:** 1 Department of Orthopaedic Surgery, University of Würzburg, 97074 Wuerzburg, Germany; 2 Department of Trauma, Hand and Reconstructive Surgery, University of Rostock, 18057 Rostock, Germanythomas.mittlmeier@med.uni-rostock.de (T.M.); 3 Institute for Experimental Surgery, University of Rostock, 18057 Rostock, Germany; 4 Olympic Training Center Mecklenburg-Vorpommern, 18057 Rostock, Germany; 5 Department of Trauma and Reconstructive Surgery, Eberhard-Karls-University Tuebingen, BG Unfallklinik, 72076 Tuebingen, Germany; 6 Institute of Sport Science, University of Rostock, 18057 Rostock, Germany; 7 Institute of Immunology, Rostock University Medical Center, 18057 Rostock, Germany; 8 Institute for Clinical and Experimental Surgery, University of Saarland, 66123 Homburg, Germany

**Keywords:** osteoporosis, muscle, force, bone, micro-CT, training

## Abstract

Testosterone deficiency in males is linked to various pathological conditions, including muscle and bone loss. This study evaluated the potential of different training modalities to counteract these losses in hypogonadal male rats. A total of 54 male Wistar rats underwent either castration (ORX, *n* = 18) or sham castration (*n* = 18), with 18 castrated rats engaging in uphill, level, or downhill interval treadmill training. Analyses were conducted at 4, 8, and 12 weeks postsurgery. Muscle force of the soleus muscle, muscle tissue samples, and bone characteristics were analyzed. No significant differences were observed in cortical bone characteristics. Castrated rats experienced decreased trabecular bone mineral density compared to sham-operated rats. However, 12 weeks of training increased trabecular bone mineral density, with no significant differences among groups. Muscle force measurements revealed decreased tetanic force in castrated rats at week 12, while uphill and downhill interval training restored force to sham group levels and led to muscle hypertrophy compared to ORX animals. Linear regression analyses showed a positive correlation between bone biomechanical characteristics and muscle force. The findings suggest that running exercise can prevent bone loss in osteoporosis, with similar bone restoration effects observed across different training modalities.

## 1. Introduction

Hypogonadism, also known as “Testosterone Deficiency Syndrome”, is a condition characterized by insufficient testosterone production in males. The etiology of hypogonadism can be attributed to various factors, including testicular damage, pituitary/hypothalamic dysfunction, genetic anomalies, inflammatory diseases, cancer treatment, aging, and obesity [1]. Hypogonadism has a considerable socioeconomic impact due to its association with various comorbidities, like type 2 diabetes mellitus, cardiovascular diseases, and osteoporosis, which, in turn, can increase healthcare costs and present significant challenges for affected individuals [2]. The global prevalence of testosterone deficiency in males ranges from 10 to 40% [3]. Furthermore, it is projected that the incidence of male hypogonadism will continue to rise [2].

The clinical symptomatology of hypogonadism in the musculoskeletal system is divergent [4,5], leading predominantly to a decline in bone mineral density and muscle mass reduction [6]. While testosterone supplementation is a substantial therapy for male hypogonadism, various research groups are exploring the use of nonpharmacological interventions, such as exercise, for managing testosterone deficiency [7].

It is widely accepted that exercise has positive effects on bone and muscle health, while a sedentary lifestyle is associated with lower bone and muscle mass [8]. Previous research has shown that in nonpathologic conditions, different exercise types impact bone shape and bone microarchitecture [9]. Furthermore, the mechanical properties of the bone are determined by the tensile muscle forces generated during muscle contraction [10]. As a result, the muscle−bone unit can be impacted differently by various types of muscle contractions. Uphill running primarily involves concentric muscle contractions [11], while downhill running, on the other hand, mostly involves eccentric muscle contractions [12]. It is assumed that downhill running is a potent osteogenic stimulus [13], whereas both uphill and downhill running can positively affect muscle force [14]. However, the optimal exercise type for bone and muscle health remains unclear [15], as does the impact of training on the peripheral skeletal muscles and bones of hypogonadal males.

In this study, we hypothesized that distinct training modalities, including uphill, downhill, and level running, would have varying effects on bone and muscle, potentially reversing muscle and bone loss in hypogonadal male rats.

## 2. Materials and Methods

### 2.1. Experimental Setup and Groups

In our study, we used 54 male Wistar rats (weighing 375–425 g and aged 12 weeks; sourced from Charles River Laboratories, Research Models and Services, Sulzfeld, Germany). The rats were housed at our research facility (Institute for Experimental Surgery, Rostock, Germany) under a 12-h day/night cycle, a constant temperature of 22 °C, and 51% relative humidity while being provided with unrestricted access to food and water.

After a week of acclimatization in the laboratory environment, the rats were randomly assigned to the experiment. At day 0, surgical castration was performed on 36 animals (ORX) and a sham castration on 18 animals (sham). The final analysis was performed at the 4th, 8th, and 12th week after the castration or the sham castration (*n* = 6 animals per group; groups: 4 wk-sham, 8 wk-sham, 12 wk-sham, 4 wk-ORX, 8 wk-ORX, and 12 wk-ORX). During this period, all sham-castrated animals and the 18 castrated animals were kept separately in their cages without any workouts.

The residual 18 castrated animals underwent uphill, level, or downhill interval training for 12 weeks after surgery (as described in the “training protocol”). A final analysis was performed on these animals at the 12th week after castration (*n* = 6 animals per group; groups: 12 wk-ORX-up, 12 wk-ORX-level, and 12 wk-ORX-down). During the final analysis, the muscle force of the left soleus muscle was measured in vivo, and tissue samples were harvested for ex-vivo analysis (whole blood samples, left soleus muscle, and both tibial bones). The experiment was concluded by euthanizing all animals by deepening the anesthesia.

### 2.2. Castration and Sham Castration

Castration and sham castration were conducted under 110 mg/kg Ketamin and 7 mg/kg Xylazin anesthesia (Ketamin, Bela-Pharm, Vechta, Germany and Xylazinhydrochlorid, Bayer Vital, Leverkusen, Germany). The surgery was performed in a supine position. The testes and surrounding area were shaved using electric clippers (Klaus Effenberger, Medizinische Geräte, Pfaffing, Germany) and disinfected using povidone-iodine (Betaisodona^®^, Mundipharma, Limburg, Germany). A straight craniocaudal incision 2 cm in length over the middle septum of the scrotum was performed using a scalpel (Präzisa plus, P.J. Dahlhausen & Co., Cologne, Germany). After dissecting the ligamentum caudae epididymidis from its insertion from the tunica dartos, the testis was mobilized. The testis was freed from the scrotum using forceps and scissors. Two ligatures were tied tightly around the spermatic cord using Vicryl ligatures (Vicryl^®^ 3–0, Johnson & Johnson, St-Stevens-Woluwe, Belgium) to perform the castration. The spermatic cord was then dissected between the ligatures. The procedure was repeated for the contralateral testis.

For the sham castration, the spermatic cord was mobilized, and the ligatures were placed around the spermatic cord without damaging it and then immediately removed. The incision was closed in layers using a Vicryl (3–0) suture and then disinfected with povidone-iodine.

After surgery, all animals received analgesia with metamizole sodium (Novaminsulfon-ratiopharm^®^, Ratiopharm, Ulm, Germany, 200 mg/kg body weight per os in drinking water) for 3 days.

### 2.3. Training Protocol

**Figure 1:**
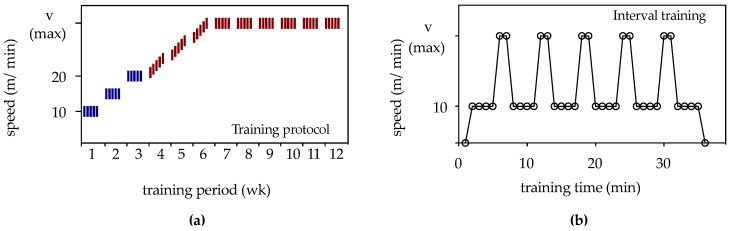
(**a**) The training protocol for the 12-week period is schematically represented. Blue lines indicate continuous training, while red lines indicate interval training. (**b**) Schematic representation of the interval training program for one rat. Each interval consisted of 7 min, with the rats running for 4 min at a speed of 10 m/min. Within 30 s, the speed was continuously increased to the maximum speed (Vmax), which was maintained for 2 min, followed by a 30-s decrease back to 10 m/min. The maximum speed varied according to the group: 28 m/min for the uphill group (12 wk-ORX-up), 35 m/min for the level group (12 wk-ORX-level), and 34 m/min for the downhill group (12 wk-ORX-down). This interval was repeated five times.

Animals from the 12 wk-ORX-up, 12 wk-ORX-level, and 12 wk-ORX-down groups underwent 12 weeks of running training. The rats ran on a treadmill specifically designed for rats (TSE Treadmill—Modular Treadmill for Rats, TSE Systems GmbH, Bad Homburg, Germany) either uphill (inclination +10°; group: 12 wk-ORX-up), level (inclination 0°; group: 12 wk-ORX-level), or downhill (inclination −10°; group: 12 wk-ORX-down). Animal training was performed 5 days per week (Monday to Friday) for 12 weeks. The 12 weeks of running training were divided into 3 phases, as shown in Figure 1:

“Learn to train” phase (weeks 1–3): During this phase, the rats were acclimatized to the treadmill. They underwent continuous training for 20 min at a speed of 10 m/min in week 1, 30 min at a speed of 15 m/min in week 2, and 40 min at a speed of 20 m/min in week 3.

“Train to train” phase (weeks 4–6): In the fourth week, the maximum speed for each group was determined during interval training (Figure 1). Starting with a running speed of 20 m/min on the first day of week 4, the speed was increased by 1 m/min each day until a maximum speed was reached. The maximum speed for each group varied based on an individual rat’s running behavior, resulting in a maximum speed of 28 m/min for the uphill group (12 wk-ORX-up), 35 m/min for the level group (12 wk-ORX-level), and 34 m/min for the downhill group (12 wk-ORX-down) by the end of week 6.

“Training” phase (weeks 7–12): In the remaining 6 weeks, the rats underwent interval training at their respective maximum speeds determined during the “Train to train” phase.

### 2.4. Muscle Force Measurement

The force generated by the left soleus muscle was indirectly assessed through the stimulation of the left sciatic nerve. A 3-cm posterolateral longitudinal incision was made on the left hind leg to access the soleus muscle. Following this, the muscle fascia was opened, and the gastrocnemius/soleus complex was carefully separated. The distal portion of the soleus muscle and the Achilles tendon were then exposed, with the Achilles tendon being detached from the calcaneus. Special care was taken during the procedure to preserve the neurovascular structures supplying the soleus muscle, ensuring its functionality during force measurements.

The left knee and ankle were positioned in the force measurement device (NW-01; Experimetria, Budapest, Hungary), and the Achilles tendon was sutured to a force sensor (FSG-01; Experimetria) using the Kirchmayr−Kessler technique (3–0 Vicryl). To access the sciatic nerve, a 1.5-cm lateral incision was made on the skin of the thigh’s middle third. The muscle fascia was then incised, and the left sciatic nerve was carefully exposed between the femur and flexor muscles. An electrode was connected to the exposed sciatic nerve, and bipolar stimulation was applied using a control unit (CRS-ST-02-O; Experimetria, Budapest, Hungary).

For determining the twitch force of the soleus muscle, bipolar stimulation was applied to the sciatic nerve at 9 mA/75 Hz. This involved five 0.1-s pulses with 5-s pause intervals between each pulse. To measure the tetanic muscle force, the soleus muscle was stimulated similarly at 9 mA/75 Hz, but with pulse durations of 3 s per stimulation and 5-s pause intervals between each pulse. The analog signal was subsequently digitized using a modem (LabJack U12, LabJack Corporation, Lakewood, CA, USA), and the recorded values were stored using a custom Visual Basic application (Visual Basic 6, Microsoft Deutschland GmbH, Munich, Germany).

### 2.5. X-ray Micro-Computed Tomography (µCT)

The left tibia was used for the µ-CT analysis. Prior to scanning, the tibia was stored in 0.9% saline solution overnight. Scans were performed with a Skyscan 1076 in vivo µ-CT (Bruker, Antwerp, Belgium). The analysis included cortical and trabecular bone morphology and bone mineral density. All scans were performed with an isotropic voxel size of 9 μm and a 0.5 mm aluminum filter at 71 kilovolts and 154 microamperes. Additional settings were a rotation step of 0.6°, frame averaging of 3, and rotation of 18°. Subsequent reconstructions were performed using the provided NRecon software (Bruker) with ring artifact reduction set to 6, defect pixel masking set to 20%, and beam hardening set to 30%.

The region of interest (ROI) for trabecular bone was manually marked in the metaphysis. The starting point for the trabecular bone analysis was 160 slices below the epiphyseal growth plate and continued for 450 slices. The cortical bone ROI in the diaphysis was also hand marked. The starting point for the cortical bone analysis was 770 slices below the epiphyseal growth plate and continued for an additional 200 slices. Bone mineral density was calculated by analyzing 2 hydroxyapatite phantoms of known mineral density (0.25 g/cm^3^ and 0.75 g/cm^3^). Final 2D and 3D analysis was performed using the CT Analyzer (Bruker, Antwerp, Belgium).

For quantification of cortical bone morphology, the following parameters were obtained using µ-CT software (Bruker): mean total bone cross-sectional area (B.Ar), mean total bone cross-sectional area (T.Ar), B.Ar/T.Ar ratio, and bone mineral density (BMD). Bone volume (Bv), total volume (Tv), bone volume fraction (Bv/Tv), bone mineral density (BMD), trabecular number (Tb.N), trabecular thickness (Tb.Th), trabecular separation (Tb.Sp), and Degree of anisotropy (DA) were used as parameters for trabecular bone morphology by µCT.

### 2.6. Biomechanical Analysis for the Tibia

For a biomechanical analysis of the bone, the right tibia was employed. Bone stiffness was assessed through a bending test using a 3-point bending device (Mini-Zwick Z 2.5, Zwick GmbH, Ulm, Germany). The tibia was oriented with its anterior surface facing upwards, and both the proximal and distal metaphyses were positioned on 2 metal supports spaced 26 mm apart. This arrangement ensured a stable and flat placement of the bone on the supports. A metal impactor was then applied to the center of the tibial shaft at a consistent speed of 10 mm/min until the bone fractured. Bending stiffness (N/mm) and ultimate load (N) were calculated from the linear elastic portion of the load-displacement diagram utilizing testXpert software (Zwick GmbH, Ulm, Germany, version 12.0).

### 2.7. Collagen Deposition

Collagen content in skeletal muscle tissue was quantified using 4 µm muscle sections stained with Sirius Red (Direct Red 80; Aldrich, Toronto, ON, Canada). The entire muscle preparation was digitally imaged at a 40× magnification using cell^D software (v 2.2; Olympus Soft Imaging Solutions GmbH, Hamburg, Germany). Subsequently, digital image manipulation was performed with an image processing program (Photoshop CS4 version 11.0.2, Adobe Systems Europe, Uxbridge, UK) to isolate collagen fibers from the remaining image structures. The collagen fiber pixel count was determined planimetrically and expressed as a percentage of the total object field.

### 2.8. Muscle Fiber Diameter

The sections were stained with hematoxylin-eosin and analyzed under a light microscope at a magnification of 200× to determine the muscle fiber diameter. The diameter of the muscle fiber in 4 μm was measured at 2 points along each fiber present in the field of view, and the average value was calculated from the 2 measurements. A total of 40 fields of view were randomly selected and measured in a meandering pattern for each section, resulting in 40 measurements per section (further details in [16]).

### 2.9. Muscle Fiber Coverage

For the evaluation of the muscle tissue fraction, the hematoxylin-eosin stained muscle sections were digitally captured in their entirety at a 40× magnification (cell^D). Using digital image processing (Photoshop), the muscle fibers were manually isolated from the remaining tissue structures, such as collagen fibers, blood vessels, or adipose tissue. Subsequently, the pixel count of the muscle fibers was calculated planimetrically and expressed as a percentage of the total object field (further details in [16]).

### 2.10. Serum Testosterone Enzyme-Linked Immunosorbent Assay (ELISA)

Upon completion of each experiment, blood samples were collected from the heart by direct puncture of the left ventricle using serum syringes. The samples were then centrifuged (GS-6R Centrifuge, Beckman Coulter, Fullerton, CA, USA) at 200× *g* and room temperature for 10 min, followed by serum storage at −20 °C. Testosterone concentrations in the serum were measured utilizing an ELISA reader (Victor3 ^TM^ X3, Perkin Elmer Inc., Waltham, MA, USA) and the corresponding enzyme immunoassay kit (Testosterone ELISA Kit; ab108666; Abcam Ltd., Cambridge, UK).

### 2.11. Statistical Analysis

The data were presented as means ± standard error (SD). Normality tests were performed for each group before statistical analysis. When the normality test was passed, differences between groups were evaluated using one-way ANOVA, followed by the Holm-Sidak method for pairwise multiple comparisons. If the normality test failed, Kruskal−Wallis one-way ANOVA on ranks was performed, followed by the Tukey test for pairwise multiple comparisons.

A linear regression analysis was employed to create the correlation matrix. When the *p*-value was significant, it indicated that Pearson’s r correlation coefficient was non-zero, suggesting the presence of a linear relationship between the two variables.

Statistical significance was considered at *p <* 0.05. Statistical analyses were conducted using the R-based open-source Jamovi software (The Jamovi project; Jamovi version 2.1; retrieved from https://www.jamovi.org, accessed on 13 April 2023). Data illustration was performed using the software Datagraph (v. 5.11; Visual Data Tools; Chapel Hill, NC, USA).

## 3. Results

### 3.1. General Observation

Upon recovery from anesthesia, all animals that underwent castration or sham castration did not experience any surgical complications. Furthermore, no signs of infection, discomfort, or illness were observed in the animals.

### 3.2. Serum Testosterone

The analysis of serum testosterone revealed a substantial decline below the ELISA detection threshold in all castrated animals (<0.2 ng/mL for groups 4 wk-ORX, 8 wk-ORX, 12 wk-ORX, 12 wk-ORX-up, 12 wk-ORX-level, and 12 wk-ORX-down). In contrast, sham-castrated animals exhibited no significant differences in serum testosterone levels across time points and groups (4-week-sham: 2.7 ± 0.8 ng/mL, 8-week-sham: 2.4 ± 0.9 ng/mL, 12-week-sham: 2.4 ± 0.7 ng/mL).

### 3.3. Bone

**Figure 2:**
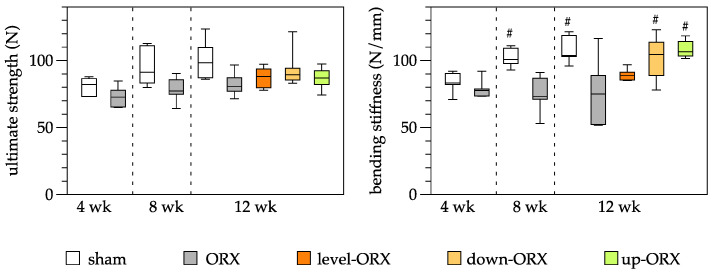
Quantitative analysis of the ultimate strength (N) and the bending stiffness (N/mm) of the tibia. The animals underwent either sham-castration (sham) or castration (ORX), accompanied by level (ORX-level), downhill (ORX-down), or uphill (ORX-up) training for 12 weeks (*n* = 6 animals per group). The data are presented as box plots (median, 1st, and 3rd quartile) with whiskers (minimum and maximum values). Statistical analysis: one-way ANOVA; # *p* < 0.05 vs. ORX.

Biomechanical analysis of the tibia revealed no significant differences in ultimate stiffness among time points and groups, with mean values spanning from 73 to 101 N. In contrast, substantial differences were observed in the bending stiffness of the tibia. The ORX group displayed significantly lower values of bending stiffness at 8 and 12 weeks following castration when compared to the sham group. Moreover, 12 weeks after surgery, both the down-ORX and up-ORX groups exhibited significantly higher values than the ORX group, and these values were akin to those of the sham group (Figure 2).

The µCT analysis of cortical bone showed no significant differences in the Bone Area to Total Area ratio (B.Ar/T.Ar) across groups and time points, with values ranging from 65.8 ± 2.2 (4-week ORX) to 72.33 ± 2.58 (12-week ORX). On the other hand, the trabecular bone parameters demonstrated diverse results. The Bone volume to Total volume ratio (Bv/Tv) had a significant decrease in the 8-week ORX group (11.17 ± 1.36) compared to the sham group, with overall values spanning from 11.11 ± 6.03 (12-week ORX) to 21.66 ± 2.48 (8-week Sham). The Trabecular number (Tb.N) exhibited a significant decline in the 8-week ORX group (0.94 ± 0.11) compared to the sham group, with values ranging between 0.95 ± 0.52 (12-week ORX) and 1.87 ± 0.17 (8-week Sham). Trabecular thickness (Tb.Th) and Degree of anisotropy (DA) remained relatively consistent across all groups and time points. Trabecular separation (Tb.Sp) showed a significant increase in both the 8-week ORX (1.14 ± 0.19) and 12-week ORX (1.21 ± 0.44) groups compared to their respective sham groups, with values varying from 0.43 ± 0.05 (8-week Sham) to 1.21 ± 0.44 (12-week ORX). The Structure model index (SMI) demonstrated a significant increase (2.02 ± 0.13) and Connectivity density (Conn.D) indicated a significant decrease in the 8-week ORX group (21.92 ± 3.46) compared to the sham group (Table 1).

**Figure 3:**
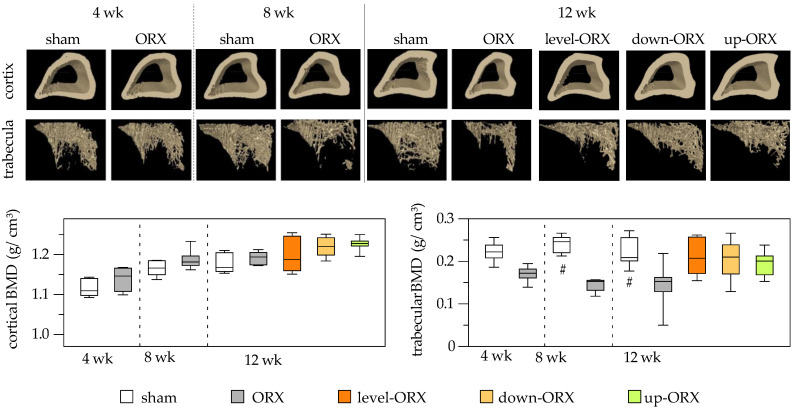
Bone mineral density (g/cm^3^) of the cortex of the tibia. On the upper side of the figure, representative micro-CT images are displayed. On the bottom side, a quantitative analysis is presented. The animals underwent either sham-castration (sham) or castration (ORX), accompanied by level (ORX-level), downhill (ORX-down), or uphill (ORX-up) training for 12 weeks (n = 6 animals per group). The data are presented as box plots (median, 1st, and 3rd quartile) with whiskers (minimum and maximum values). Statistical analysis: one-way ANOVA; # *p <* 0.05 vs. ORX.

No differences were observed in cortical BMD between time points and groups. However, for trabecular BMD, ORX animals exhibited significantly lower values compared to sham-operated animals at 8 and 12 weeks postcastration. Engaging castrated rats in uphill, level, and downhill training for 12 weeks led to a slight increase in trabecular BMD compared to sedentary animals. Importantly, no significant differences were found among the sham, level-ORX, up-ORX, and down-ORX groups 12 weeks after castration (Figure 3).

### 3.4. Muscle

**Figure 4:**
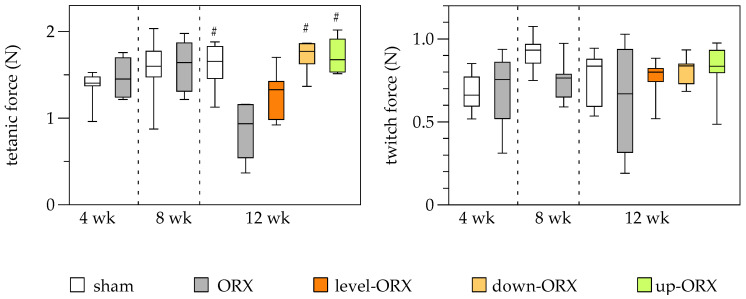
Quantitative analysis of the tetanic force (N) and the twitch force (N) of the soleus muscle. The animals underwent either sham-castration (sham) or castration (ORX), accompanied by level (ORX-level), downhill (ORX-down), or uphill (ORX-up) training for 12 weeks (*n* = 6 animals per group). The data are presented as box plots (median, 1st, and 3rd quartile) with whiskers (minimum and maximum values). Statistical analysis: one-way ANOVA; # *p <* 0.05 vs. ORX.

Muscle force measurements presented no significant differences in tetanic force between sham and ORX groups on both week 4 and day 8 after ORX surgery. However, by week 12, the ORX group showed a significant decrease in muscle force compared to the sham group. Notably, orchiectomized animals that underwent downhill and uphill exercise training demonstrated restoration of tetanic force to levels similar to those in the sham group. Throughout the study, no significant differences were observed in twitch force measurements across groups and timepoints (Figure 4).

**Figure 5:**
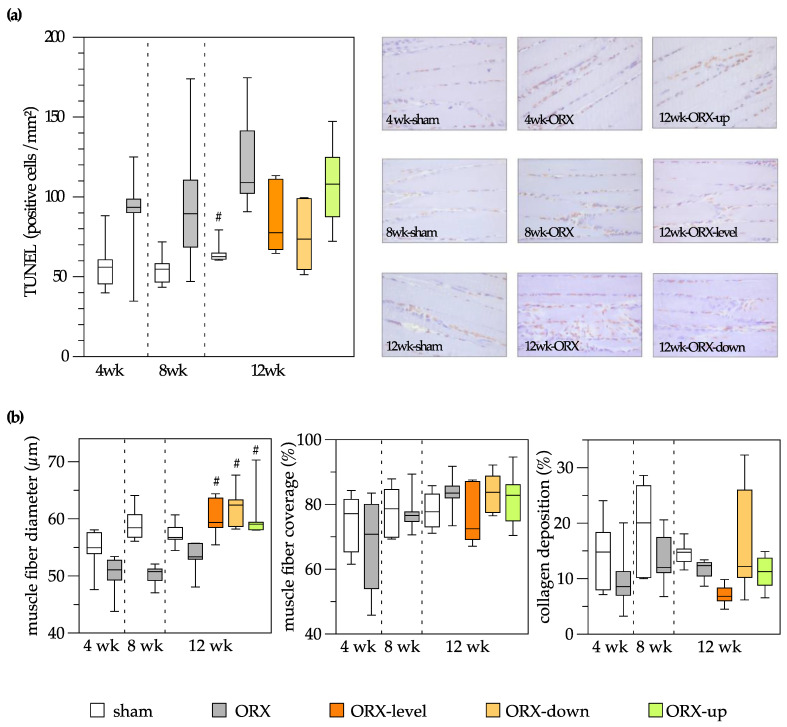
On the left side of the figure, a quantitative analysis is presented, and on the right side, representative images are displayed (400× magnification). Apoptosis of the soleus muscle was assessed through analysis of TUNEL-positive cells (cells/mm²) (**a**). Quantitative analysis of the muscle fiber diameter (µm), muscle fiber coverage (%), and collagen deposition (%) of the soleus muscle (**b**). The animals underwent either sham-castration (sham) or castration (ORX), accompanied by level (ORX-level), downhill (ORX-down), or uphill (ORX-up) training for 12 weeks (n = 6 animals per group). The data are presented as box plots (median, 1st, and 3rd quartile) with whiskers (minimum and maximum values). Statistical analysis: one-way ANOVA; # *p <* 0.05 vs. ORX.

Histological and immunohistochemical analyses demonstrated a decrease in muscle fiber diameter and an increase in apoptotic muscle cells after orchiectomy. Treadmill training (comprising uphill, level, and downhill training) effectively counteracted the reduction in muscle fiber diameter induced by orchiectomy, resulting in values surpassing those observed in the sham control group. Furthermore, 12 weeks of treadmill training in orchiectomized rats did not affect muscle cell apoptosis. No significant differences were detected between time points and groups concerning muscle fiber coverage and collagen deposition (Figure 5).

### 3.5. Bone and Muscle

To investigate the potential influence of muscle force on the biomechanical characteristics of the bone, linear regression analyses were conducted. The results showed a significant positive linear correlation between the ultimate strength and bending stiffness of the tibia and both the tetanic force and twitch force of the soleus muscle. Additionally, a significant positive linear correlation was found between the bending stiffness of the tibia and the twitch force of the soleus muscle (Table 2).

## 4. Discussion

The pathophysiology underlying the effects of testosterone and testosterone deficiency in males on muscle and bone tissue exhibits numerous parallels. Testosterone levels exert both direct and indirect effects on bone metabolism. Orchiectomy-induced testosterone deficiency has been shown to contribute to osteoporosis in rat models by reducing bone formation, primarily through osteoblast activation [17]. As testosterone levels decrease, osteoblast activity declines, resulting in diminished bone formation. Orchiectomy-induced testosterone decline can also enhance osteoclast activity, leading to increased bone resorption and loss of bone mass [18]. Furthermore, testosterone indirectly influences bone metabolism by modulating the levels of other hormones and proteins, such as estrogen, parathyroid hormone, and insulin-like growth factor 1 [19]. A similar pathophysiological mechanism has been identified in muscle tissue [20]. Both testosterone and mechanical loading contribute to the upregulation of muscle insulin-like growth factor-1 (IGF-1), an essential regulator of muscle mass, through the activation of protein kinase B (Akt). Investigations involving the selective ablation of androgen receptors in myocytes have demonstrated a decrease in muscle IGF-1 expression. In men, testosterone deficiency has been associated with a reduction in both circulating and intramuscular IGF-1 expression. Corresponding effects have been observed in orchiectomized rodent models. Consequently, it was expected that our findings would demonstrate the training’s ability to facilitate the restoration of both musculature and bone health within hypogonadal male rats.

The current study reveals that castration induces osteoporotic effects on the tibia of Wistar rats, particularly in the trabecular bone, in a relatively short period. The literature supports our findings, as osteoporosis has been shown to occur in rats around eight weeks after orchiectomy [21]. Numerous studies have examined the impact of treadmill training on osteoporotic bone, yielding mixed results. Some research groups have reported a decrease in bone mass following training, while others have observed improved bone properties postexercise. Ip et al. [22] suggested that moderate training (0° inclination; 20 m/min for 1 h; 5 days/week for 9 weeks) in obese rats led to weight reduction but did not increase bone mass. The authors postulated that more intensive training might affect bone mass. Joo et al. [23] conducted an endurance exercise study and found that exercised animals (0° inclination; 30 m/min for 1 h; 5 days/week for 10 weeks) exhibited increased bone mineral density, bone volume, bone volume fraction, trabecular thickness, and trabecular number. Additionally, the trabecular bone pattern factor was significantly lower compared to sedentary controls. These findings align with our observations, suggesting that intensive training is associated with increased ground reaction forces, which are essential for positive bone responses [24]. Warner et al. [25] compared the trabecular and cortical bone properties of the femur after ovariectomy, treadmill training (15° inclination; 17 m/min for 1 h; 5 days/week for 12 weeks), and swimming (60 min; 5 days/week for 12 weeks). They did not observe any significant differences between the groups, indicating that training intensity is a critical factor for bone regeneration. Other groups demonstrated that intensive jumping exercises in ovariectomized animals restored the bone mineral density and mechanical properties of the bone to a control level [26].

Bone regeneration can be positively impacted by mechanical stimulation, as evidenced by various studies. Hagihara et al. [27] demonstrated that treadmill training enhances the bone mineral density of the femur and tibia, while no significant changes were observed in the less load-bearing second lumbar vertebral body. Furthermore, eccentric muscle training has been shown to be more osteogenic than concentric muscle training [28,29]. Consequently, it is plausible to infer that uphill and downhill exercises may induce a more pronounced enhancement of bone properties compared to level training. In our study, we observed a more pronounced bone restoration in the tibia following uphill or downhill training. We hypothesize that the observed outcome on the bone could be linked to muscle atrophy caused by castration, as demonstrated in our findings and comparable studies [30]. This process likely leads to decreased tensile stress on the tibia, lessening the bone’s regenerative response to training.

The beneficial effects of training on osteoporotic patients have been reported not only in animal studies but also in numerous clinical investigations. For instance, Roghani et al. [31] described a 6-week treadmill training regimen (30 min, 3 times per week) in postmenopausal osteoporotic women that stimulates bone synthesis and reduces bone absorption. Similarly, Gunendi et al. [32] reported that a 30-min treadmill training session twice a week for 4 weeks significantly improves static and dynamic balances in postmenopausal osteoporosis patients. Additionally, a 24-week high-impact loading exercise program has been shown to increase bone mineral density and muscle strength in osteopenic postmenopausal women [33].

The literature presents varying findings on the influence of orchiectomy on physical performance during treadmill training. Some studies support the hypothesis that orchiectomy does not impact physical performance [34], while others argue that there is a notable effect on physical performance following orchiectomy [35]. In human subjects, specifically men with prostate cancer receiving androgen deprivation therapy, football training over 12 weeks improved lean body mass and muscle strength compared to usual care [36]. Additionally, 16 weeks of strength training during androgen deprivation therapy increased the type II fiber cross-sectional area and reduced the myonuclear domain in type I fibers in prostate cancer patients. Interestingly, the typical increase in satellite cell numbers observed following strength training was not seen in these patients [37].

Several studies have reported analogous outcomes in ovariectomized, aged female rats. A recent investigation specifically revealed that gradual downhill exercise could influence bone morphology and muscle quality in a manner comparable to gradual uphill training interventions during aging. It has been observed that gradual downhill running ameliorates age-related skeletal muscle and bone weakness [38]. Furthermore, interval running training has been shown to mitigate age-related skeletal muscle atrophy and bone loss in experiments conducted on ovariectomized rats [39].

In previous literature, it has been established that the primary determinant of mechanical bone properties, such as ultimate bone strength and bending stiffness, is cortical bone mineral density, followed by trabecular bone mineral density [40]. However, the present study was unable to verify this relationship directly. Notably, we observed increased cortical bone mineral density postcastration exclusively in the uphill group, which correlated with the enhanced bending stiffness in the same group. Intriguingly, the downhill training group exhibited increased bending stiffness upon castration, despite no significant alterations in cortical bone mineral density. Maurel et al. [41] reported a similar discrepancy between cortical bone mineral density and biomechanical bone properties in an analogous exercise setting involving ethanol-induced bone toxicity. We posit that biomechanical bone characteristics can undergo significant modifications due to load, even with minimal changes in bone mineral density [42]. The augmented load can lead to transformations in bone microarchitecture, ultimately serving as a critical determinant of bone stiffness and strength [43].

Our study was constrained by certain limitations, including the absence of histological or immunohistochemical analysis of the bone. Although the investigation of cellular roles within the bone postcastration and posttraining fell outside the scope of our study, we assume that regulation of the equilibrium between osteoclasts and osteoblasts led to the observed structural effect of the bone. Previous research suggests that resistance training can enhance osteoblastic activity at the endocortical surface of the bone in orchidectomized rats [44]. Notomi et al. investigated the impact of a tower climbing regimen on osteoclast behavior within the femur of rats [45]. After the experiments, in the eighth week, it was demonstrated that orchidectomized and exercised rats exhibited a decline in osteoclast surface compared to the ORX-sedentary group. Another research group suggested that initiating treadmill running immediately after orchidectomy results in an increase in bone density and calcium content in castrated rats. The primary mechanism for this observed effect was the inhibition of bone resorption, as suggested by a decrease in urinary deoxypyridinoline excretion. However, the exercise regimen they applied (60% maximal O_2_ consumption, 1 h/day, 6 days/wk for 15 wk) did not seem to have a significant impact on osteoblastic activity, as there were no observable differences in plasma osteocalcin concentrations between castrated exercising and castrated sedentary rats [46]. Additionally, it has been observed by the same group using the same experimental protocol [47] that treadmill exercise three months postorchidectomy can restore bone mineral density and bone mineral content to the baseline levels seen in untreated animals. This restoration seems to be chiefly accomplished by inhibiting bone resorption, as demonstrated by the decreased excretion of the biochemical bone marker, urinary deoxypyridinoline. Moreover, this restoration appears to occur without any concurrent reduction in osteoblastic activity, as signified by the levels of plasma osteocalcin [47]. Based on this review of existing literature, we propose that training postcastration likely enhances osteoblastic activity and decreases osteoclastic activity, culminating in reduced bone resorption.

An additional limitation of the current study is that the underlying molecular mechanisms occurring during bone regeneration and training were not investigated, although this is a goal for our group’s future research. Considering the quadrupedal running of rats, we propose that analyzing other bones, such as the femur, humerus, and radius, in the same experimental setting may provide a more comprehensive understanding of bone regeneration in relation to loading forces.

## 5. Conclusions

In summary, our study unveils the significant impact of castration on trabecular bone loss, reduced bone bending stiffness, diminished muscle force, and increased muscle cell apoptosis. Treadmill training has been identified as an effective strategy for promoting trabecular bone restoration and enhancing tetanic muscle force by counteracting muscle atrophy and partially reversing muscle cell apoptosis. In the current experimental setup, the regenerative outcomes in muscle tissue are independent of the chosen training modality. From our standpoint, treadmill training offers a promising, low-risk adjuvant therapeutic approach for mitigating the osteoporotic effects on bones.

To further advance our understanding, future research should broaden the investigative scope to explore the interplay between bone and muscle, as well as elucidate the molecular mechanisms underlying our observations.

## Figures and Tables

**Table 1:** µ-CT analysis of the tibia.

	4. Week Sham	4. Week ORX	8. Week Sham	8. Week ORX	12. Week Sham	12. Week ORX	12. Week Level-ORX	12. Week Down-ORX	12. Week Up-ORX
Cortical bone									
T.Ar	11.52 ± 0.14	10.80 ± 0.88	12.40 ± 0.44	11.09 ± 0.84	12.53 ± 0.75	11.32 ± 1.19	10.82 ± 1.09 *	11.81 ± 0.62	11.50 ± 1.19
B.Ar	7.63 ± 0.32	7.09 ± 0.39	8.33 ± 0.24	7.60 ± 0.56	8.98 ± 0.42	8.17 ± 0.7	7.80 ± 0.57 *	8.26 ± 0.68	8.02 ± 0.52 *
B.Ar/T.Ar	66.20 ± 2.24	65.80 ± 2.20	67.23 ± 0.74	68.5 ± 1.57	71.70 ± 2.42	72.33 ± 2.58	72.27 ± 2.24	69.88 ± 2.56	70.13 ± 5.13
Trabecular bone									
Bv	14.85 ± 2.09	9.05 ± 3.20	17.29 ± 2.51	6.70 ± 1.42 *	13.21 ± 3.71	7.31 ± 5.44	11.32 ± 5.26	12.03 ± 5.13	9.88 ± 3.10
Tv	74.01 ± 5.78	66.54 ± 12.91	79.64 ± 5.66	59.99 ± 10.55	71.49 ± 11.96	62.47 ± 15.96	62.81 ± 14.29	66.67 ± 10.80	61.78 ± 10.83
Bv/Tv	20.12 ± 2.79	13.27 ± 2.4	21.66 ± 2.48	11.17 ± 1.36 *	18.39 ± 4.17	11.11 ± 6.03	17.52 ± 5.13	17.49 ± 4.98	15.89 ± 3.03
Tb.N	1.77 ± 0.16	1.22 ± 0.21	1.87 ± 0.17	0.94 ± 0.11 *	1.52 ± 0.35	0.95 ± 0.52	1.45 ± 0.51	1.44 ± 0.54	1.25 ± 0.20
Tb.Th	0.12 ± 0.01	0.11 ± 0.01	0.12 ± 0.01	0.12 ± 0.01	0.13 ± 0.01	0.12 ± 0.01	0.13 ± 0.02	0.13 ± 0.02	0.13 ± 0.01
Tb.Sp	0.72 ± 0.15	1.10 ± 0.21	0.43 ± 0.05	1.14 ± 0.19 *	0.61 ± 0.17	1.21 ± 0.44 *	0.77 ± 0.34	0.78 ± 0.38	0.91 ± 0.26
DA	0.66 ± 0.03	0.68 ± 0.03	0.6 ± 0.02	0.61 ± 0.06	0.6 ± 0.05	0.59 ± 0.03	0.56 ± 0.05	0.57 ± 0.06	0.55 ± 0.08
SMI	1.43 ± 0.13	1.84 ± 0.17	1.64 ± 0.17	2.02 ± 0.13 *	1.80 ± 0.24	2.06 ± 0.31	1.78 ± 0.15	1.84 ± 0.18	1.82 ± 0.09
Conn.D	48.39 ± 4.19	31.77 ± 4.92	49.50 ± 5.12	21.92 ± 3.46 *	36.34 ± 10.99	22.97 ± 12.48	35.82 ± 16.13	31.84 ± 15.48	27.58 ± 6.18

All animals underwent either sham-castration (sham) or castration (ORX), accompanied by level (ORX-level), downhill (ORX-down), or uphill (ORX-up) training for a 12-week period (*n* = 6 animals per group). The data is presented as mean ± SD. Statistical analysis: one-way ANOVA; * *p <* 0.05 vs. sham. B.Ar: cross-sectional bone area, T.Ar: mean total cross-sectional bone area, Bv: bone volume, Tv: total volume, Tb.Th: Trabecular thickness, Tb.N: Trabecular number, Tb.Sp: Trabecular separation, DA: Degree of anisotropy, SMI: Structural model index, Conn.D: Connectivity density.

**Table 2:** Multiple linear regression analysis for biomechanical properties of the soleus muscle and the tibia.

		Twitch Force of the Soleus Muscle	Tetanic Force of the Soleus Muscle
Ultimate strength (N) of the tibia	Pearson’s r	0.259	0.275 ^+^
	*p*-value	0.064	0.046
Bending stiffness (N/mm) of the tibia	Pearson’s r	0.319 ^+^	0.477 ^+^
	*p*-value	0.021	<0.001

Pearson’s r and *p*-value are provided for the correlation between the ultimate strength and bending stiffness of the tibia and the twitch force and titanic force of the soleus muscle. The *p*-values that are below the threshold of 0.05 are indicated with ^+^.

## Data Availability

The data generated during the current study are available from the corresponding author upon reasonable request.
